# Physical Activity and Brain Health

**DOI:** 10.3390/genes10090720

**Published:** 2019-09-17

**Authors:** Carlo Maria Di Liegro, Gabriella Schiera, Patrizia Proia, Italia Di Liegro

**Affiliations:** 1 Department of Biological, Chemical and Pharmaceutical Sciences and Technologies (Dipartimento di Scienze e Tecnologie Biologiche, Chimiche e Farmaceutiche) (STEBICEF), University of Palermo, 90128 Palermo, Italy; carlomaria.diliegro@unipa.it (C.M.D.L.); gabriella.schiera@unipa.it (G.S.); 2 Department of Psychology, Educational Science and Human Movement (Dipartimento di Scienze Psicologiche, Pedagogiche, dell’Esercizio fisico e della Formazione), University of Palermo, 90128 Palermo, Italy; patrizia.proia@unipa.it; 3 Department of Biomedicine, Neurosciences and Advanced Diagnostics (Dipartimento di Biomedicina, Neuroscienze e Diagnostica avanzata) (Bi.N.D.), University of Palermo, 90127 Palermo, Italy

**Keywords:** physical activity, brain health, myokines, BDNF, Irisin, lactate, exercise and neurodegeneration, exercise and aging

## Abstract

Physical activity (PA) has been central in the life of our species for most of its history, and thus shaped our physiology during evolution. However, only recently the health consequences of a sedentary lifestyle, and of highly energetic diets, are becoming clear. It has been also acknowledged that lifestyle and diet can induce epigenetic modifications which modify chromatin structure and gene expression, thus causing even heritable metabolic outcomes. Many studies have shown that PA can reverse at least some of the unwanted effects of sedentary lifestyle, and can also contribute in delaying brain aging and degenerative pathologies such as Alzheimer’s Disease, diabetes, and multiple sclerosis. Most importantly, PA improves cognitive processes and memory, has analgesic and antidepressant effects, and even induces a sense of wellbeing, giving strength to the ancient principle of “*mens sana in corpore sano*” (i.e., a sound mind in a sound body). In this review we will discuss the potential mechanisms underlying the effects of PA on brain health, focusing on hormones, neurotrophins, and neurotransmitters, the release of which is modulated by PA, as well as on the intra- and extra-cellular pathways that regulate the expression of some of the genes involved.

## 1. Introduction

The discovery of the nervous system dates back to the ancient Greek physicians-philosophers Alcmaeon, Praxagoras, Herophilus [1,2], and Erasistratus [2]. Herophilus (c335–c280 B.C.), in particular, by dissecting human cadavers, was able to describe the structure of the brain and nerves, and to realize that motor nerves were joined to muscles, while other nerves (the sensory ones) went to organs, and were responsible for sensation. He promoted a cerebrocentric view of mind [1,2,3,4,5] and, interestingly, believed that exercise and a healthy diet were fundamental for maintaining a healthy body, and a healthy mind [3]. Over the centuries this idea has recurred many times. However, we have only recently begun to understand the cellular and molecular reasons why sedentary life is detrimental for human health, and to realize that physical activity (PA) can be a powerful medicine to counteract its effects. Actually, this is not surprising since the ability of our species to survive in many different environments, to escape predators, and to look around for food has depended on, and still depends on the ability to perform PA, and PA has thus shaped our physiology [6]. Starting from the consideration that modern humans have not only a very large brain but also a remarkable endurance capacity, it was suggested that PA also shaped our brains: It was reported, for example, that the appearance in evolution of skeletal properties related to endurance capacity correlated with the increase of brain size in hominins such as *Homo erectus* [7,8,9]. As reported by Hill and Polk [9], aerobic fitness (required for successful endurance activity), and aerobic capacity (measured as maximal oxygen consumption during exercise, VO_2_ max) correlate with brain size, both in humans and other animals; moreover, selective breeding in rodents for endurance running capacity affects both their general physiology and their brain, and also potentiates their cognitive abilities [9,10]. A further aspect of humans that might correlate with PA concerns the integumentary system: Our hairless skin indeed enhances evaporation, thus allowing dispersion of excess heat produced during endurance activity [9,11,12,13]; at the same time, a hairless skin facilitates production of vasodilatory factors, such as nitric oxide (NO), with different mechanisms [14,15].

In this context, it is important to underline that, when the importance of PA during the evolution of our species is discussed, the focus is on every movement that requires activity of our skeletal muscles, and energy expenditure. On the other hand, any planned and structured activity that is voluntarily aimed at improving and/or maintaining our physical fitness should be better defined as exercise [16]. Thus, most of the experimental work cited in this review actually concerns “exercise” since the observations reported rely on a specific series of structured, planned, and repetitive activities. Exercise is, however, only a subset of physical activity; accordingly, we will use the term “exercise” when describing the results of programmed sets of experiments, and the expression “physical activity” (PA) when discussing the effects on health of either programmed or not programmed skeletal muscle movements, in daily life.

There are clear indications that PA also has important effects on human brain health at any age and have been included, for example, in the Physical Activity Guidelines for Americans, issued by the U.S. Department of Health and Human Services (HHS) in 2018 [17,18,19]. Interestingly, in these guidelines, four classes of age, with different PA requirements, have been set: 1. Preschool-Aged Children (3–5 years)—they should be physically active throughout the day to enhance growth and development, it is also important to underline that playing develops mental capacities and social interactions in many ways; 2. Children and Adolescents (6–17 years)—they should do 60 min or more per day of moderate-to-vigorous physical activity, most of which should be aerobic, with vigorous activity for at least 3 days per week, including muscle- and bone-strengthening physical activity; 3. Adults—according to the Guidelines “Adults should move more and sit less throughout the day”. They should do at least 150–300 min of moderate-intensity PA, or 75–150 min of vigorous aerobic PA per week, together with muscle-strengthening activities of moderate-high intensity, at least 2 days a week; 4. Old Adults—they should do as much aerobic and muscle-strengthening activities as they can, on the basis of their individual health conditions. In addition, the guidelines suggest physical training for women during pregnancy and post-partum period and for adults with chronic diseases and/or disabilities [17].

PA is thus recommended as a non-pharmacologic therapy for different pathological affections as well as for the maintenance of general health status. Habitual exercise improves cardiorespiratory fitness and cardiovascular health [20,21,22,23,24], helps reducing body mass index [25,26], and can represent a natural, anti-inflammatory “drug” in chronic diseases, such as type 2 diabetes mellitus (T2DM) and cardiovascular disease (CVD) [27,28]. Moreover, given the strong association of pathologic conditions such as high blood pressure with blood–brain barrier alterations and brain dysfunctions, PA can also have beneficial effects on cerebrovascular and cognitive functions [23]. In addition, anti-depressive- [29], and analgesic-PA effects have been reported [30]. However, it has also been suggested that the anti-inflammatory effects can differ among different training programs [31], and that, while regular exercise can increase immune competence and reduce the risk of infection with respect to a sedentary lifestyle, acute and heavy bouts of activity can even have the opposite effect [27], and, in general, negative effects on health [32,33].

As discussed below, both endurance activity (i.e., long-lasting aerobic activity, such as running) and resistance exercise (i.e., exercise in which the predominant activity involves pushing against a force) have been shown to induce an increase of circulating growth factors (such as insulin-like growth factor 1, IGF-1), and neurotrophins (such as the brain-derived neurotrophic factor, BDNF) which have an effect on the brain both during development and in the adult. The same factors might have had an impact during hominin brain evolution [9], and can affect brain plasticity in the young as well as in the adult, under many different conditions, such as physiologic aging, neurodegenerative pathologies, and recovery after acute brain damage.

In this review we will discuss the putative cellular and molecular mechanisms underlying the mentioned effects of PA on the nervous system, focusing on genes known to be involved, as well as on epigenetic effects due to DNA methylation, histone post-translational modifications and exchange, and on the possible role of non-coding RNAs.

## 2. Brain Plasticity, Adult Neurogenesis, and Physical Activity

The brain capacity to adapt to ever-changing conditions, known as brain plasticity, depends on the ability of neurons to modify the strength and composition of their connections in response to both external and internal stimuli. The long-term potentiation (LTP) in synaptic efficacy constitutes the physiologic base for learning and memory. An important way for regulating neuronal function is the activity-dependent synapse-to-nucleus signalling, that can arise both in the post-synaptic and in the presynaptic element [34,35,36,37,38]. These signals are generated through different mechanisms, such as: (i) Calcium waves due to calcium-induced calcium release (CIRC) from the endoplasmic reticulum (ER) [35,39,40]; (ii) retrograde transport of proteins (e.g., **Jacob,** CREB Regulated Transcriptional Coactivator 1, **CRTC1**); Abelson-interacting protein 1, **Abi1**; the amyloid precursor protein intracellular domain associated-1 protein, **AIDA-1**; and the nuclear factor kappa-light-chain-enhancer of activated B cells, **NF-κB**); these proteins are post-translationally modified following synaptic activity, and transported to the nucleus, where they act on gene transcription, and thereafter on synaptic plasticity [34,35,36,37,38,41,42]; (iii) formation and microtubule-dependent trafficking of mRNA-protein complexes, that, after exiting the nucleus, move to neuronal periphery, where the mature transcripts localize in a repressed state, in response to local signalling, through activity-dependent activation of specific enzymes, the regulatory proteins can be then modified, for example, by phosphorylation, and the mRNAs can be translated; some of the newly synthesized proteins can accumulate at the synapse, while others can shuttle back to the nucleus to modify chromatin structure and expression [43].

By regulating synapse-to-nucleus signalling, all these events are crucial for allowing synapse activity to result in the specific gene expression programs necessary for learning and memory. In agreement with this idea, the impaired function of these signalling proteins brings about intellectual disability, psychiatric disorders, or neurodegeneration [37,38,42]. On the other hand, we can hypothesize that an increase of their function, for example as a response to PA, could also enhance brain functions and plasticity.

In the past, it was generally accepted that new neurons could not be generated in the adult to replace dying cells, and this limitation was also considered to be the main cause of neurodegeneration as well as of cognitive decline in the elderly population. However, since the 1960s, many researchers presented data suggesting that, in all the mammals analysed, new neurons could be generated in the sub-granular zone (SGZ) of the dentate gyrus of the hippocampus, and in the sub-ventricular zone (SVZ) of the lateral ventricles, in the postnatal and adult life [44,45,46,47,48,49,50]. In particular, neurons born in the SGZ were shown to differentiate and integrate into the local neural network of the hippocampus. These findings are extremely important since the hippocampus is fundamental for the formation of certain types of memory, such as episodic memory and spatial memory [51,52,53,54]. In addition, hippocampus-dependent learning is one of the major regulators of hippocampal neurogenesis [55]: living in environments which stimulate learning enhances, in rats, the survival of neurons, born in the adult from neural stem cells (NSCs) [52].

**Figure 1:**
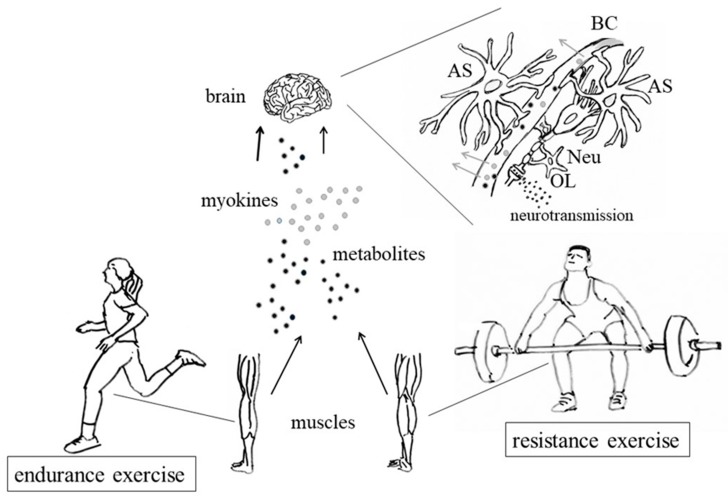
Hypothetical pathway for the exercise-mediated effects on brain functions: both endurance and resistance exercise, even if with different kinetics and properties, allow muscle synthesis, and release myokines (e.g., brain-derived neurotrophic factor, BDNF), as well as of metabolites (such as lactate) into the circulation; these molecules can cross the blood­­­­–brain barrier (BBB) at the level of the brain capillaries (grey arrows) and affect the functions of both neurons and glial cells, thus modifying neurotransmission in different regions of the brain. As explained in the text, neurotransmission can then activate pathways leading to modifications of gene expression. AS: astrocytes; BC: brain capillaries; Neu: neurons; OL: oligodendrocytes.

Now, increasing evidence suggests that PA, largely due to factors released by contracting muscles (Section 3; Figure 1), can improve brain functions, such as memory and attention, in both children and adults [56,57,58,59,60,61,62,63,64]. A few examples of single studies (first three rows) and reviews/meta-analyses (second three rows), aimed at ascertaining any relationship between PA and learning/memory, are given in Table 1.

The data reported in Table 1 clearly indicate that PA has a positive effect on mental health and abilities, especially in adolescents; however, as reported in the “Conclusions” column (sentences in bold letters), most authors agree on the fact that the previous studies do not yet give uniform indications on the relationships between the type/intensity/frequency of exercise and the brain health outcomes; these limitations derive, on one hand, from the wide range of conditions set in the exercise programs, and on the other hand, the differences from study to study also depend on the variability of the parameters chosen to evaluate mental health. We also have to add to these considerations the poor knowledge we still have of ‘mind’ and of ‘mental health’. Thus, many laboratories are now focusing on exercise-dependent cellular and molecular modifications of brain cells activity, in the attempt to uncover the mechanisms underlying PA–mental health biochemical relationships.

At the cellular level, it was found that treadmill exercise can increase hippocampal neurogenesis in aged mice [68]. Interestingly, exercise can also affect the proliferation [69,70], as well as size and function, of astrocytes [71]. These latter events regulate, in turn, the number and localization of neuronal synapses, and might influence LTP and episodic memory formation [72].

Many researchers suggested that all these effects are also regulated by the brain capillaries (BC, Figure 1) that reach the neurogenic niche, supplying angiogenetic growth factors, such as the growth and differentiation factor 11 (**GDF11**), the vascular endothelial growth factor (**VEGF**) [59], and **BDNF**, that activates a cellular survival pathway involving the serine-threonine kinase **AKT** and **CREB**, thus inducing the transcription of genes responsible for almost all the aspects of neuroplasticity [59,72]. The neurogenic niche also receives axonal inputs from both local and distant neurons, which release a variety of neurotransmitters, such as serotonin, glutamate, and GABA [59]. For example, glutamate, through interaction with NMDARs, is thought to regulate LTP in response to exercise [73]. Many epidemiologic studies, mostly in the last two decades, also revealed a link between PA, human brain health (and longevity) and epigenetic modifications of the genome, even leading, on one hand, to the concept of “epigenetic age” or “DNA methylation age” (essentially measured, however, as blood cells DNA methylation) [74,75,76,77,78], and, on the other hand, to the acknowledgment that epigenetic mechanisms induced by PA can build up an “epigenetic memory” that affects long-term brain plasticity, neurogenesis, and function [79,80,81,82]. Intriguingly, it has been proposed that epigenetic modifications caused by lifestyle and diet, as well as the effects of PA can be heritable (discussed in [83]).

Epigenetic processes modify eukaryotic chromatin structure, and hence gene expression, without changing the underlying DNA sequence, through at least three mechanisms: (i) DNA methylation/demethylation, and post-translational modifications (such as methylation/demethylation and acetylation/deacetylation), of histones on specific residues of their N-terminal tails; (ii) substitution of some histone isotypes with other histone variants; (iii) sliding and/or removal of the basic chromatin structural organization elements (nucleosomes), due to specific ATP-dependent chromatin remodelling complexes [84,85,86,87]. Specific proteins are then able to “read” and bind DNA and histone tail modifications, thus creating synergic complexes which can activate or depress transcription [88,89,90,91,92]. Importantly, in some of these remodelling events, long noncoding RNAs (lncRNAs) also play a role [93]. Finally, gene expression can be regulated by short noncoding RNAs, called microRNAs (miRNAs), which are able to pair with sequences mainly present in the 3′-UTR of their target mRNAs, thus inducing inhibition of their translation or even their degradation [94,95,96].

In summary, while the genome of an organism is relatively stable over the lifespan, its expression (i.e., the phenotype) is influenced by many epigenetic factors. Most important, we now know that inactivity is epigenetically deleterious: for example, it has been reported that nine days of bed rest can induce insulin resistance in otherwise healthy subjects. The analysis of the pathways affected revealed a significant downregulation of 34 pathways, mainly involving genes associated with the mitochondrial function, including the peroxisome proliferator-activated receptor γ co-activator 1α (PPARGC1A, or **PGC-1α**). An increase of PPARGC1A DNA methylation was also reported, and this epigenetic modification was not completely reversed after four weeks of retraining, thus highlighting the importance of daily physical activity [76,97].

### 2.1. Brain-Derived Neurotrophic Factor (BDNF)

BDNF is a neurotrophin involved in all the most important aspects of neuroplasticity, from neurogenesis to neuronal survival, from synaptogenesis to cognition, as well as in the regulation of energy homeostasis.

Both in humans and rodents, the *BDNF* gene contains nine exons, each of which has its own promoter. As a result of this gene structure, many species of mature transcripts are known, even if the final translation product is the same for all of them [98,99]. The existence of different promoters, however, is important in terms of temporal and spatial regulation, including the possibility that different promoters are used in different cell types and brain regions [99].

In the published literature, a generalized exercise-dependent increase of BDNF has been reported. A few examples of both single studies (first six rows) and reviews/meta-analyses (last two rows) aimed at ascertaining PA effects on BDNF levels are reported in Table 2.

The BDNF increase seems to correlate with the exercise volume (given by “intensity + duration + frequency” of activity) [100]. However, it was also reported that the greatest responses are given by well-trained individuals, while mainly sedentary subjects show lower or even no response [100,101]. Interestingly, open-skill exercise (e.g., badminton) increases BDNF levels more than closed-skill exercise (e.g., running), probably because open-skill activities require additional attention to face ever-changing situations [102], and possibly also because they are more enjoyable.

As a whole, data reported in Table 2 indicate an exercise-dependent BDNF increase. Again, as evident in the “Conclusions” column (sentences in bold letters), however, a great variability emerges from the different studies.

In general, BDNF increase seems to correlate with increased catabolic requirements, and with a higher production of reactive oxygen species (ROS), as a consequence of the increased mitochondrial activity. Then, in the brain, BDNF stimulates mitochondrial biogenesis, and acts as a metabotrophin to mediate the effects of exercise on cognition [109,110].

Actually, *BDNF* gene transcription does not depend on a single regulatory pathway: it is synergistically stimulated by a complex array of factors, some of which, as discussed above, reach the nucleus only when neurons are active. In addition, the already mentioned transcription factor coactivator **PGC-1α** increases sharply under energy-requiring conditions, both in muscles (see Section 3) and neurons, and contributes to raising BDNF levels [100].

Notably, the expression of the *BDNF* gene is also controlled at the epigenetic level. In 2006, Tsankova et al. [111] analysed the effects of a chronic social defeat stress on the *BDNF* gene chromatin organization in the mouse hippocampus, and found that stress induced a lasting downregulation of BDNF transcripts III and IV, as well as an increase in both histone and promoter methylation. The stressing protocol was followed by treatment with an antidepressant that reversed these effects, also inducing histone acetylation and downregulation of histone deacetylase (HDAC) 5 [111]. Starting from these results, in 2011, Gomez-Pinilla et al. [112] studied the epigenetic effects of exercise on BDNF chromatin regulation, and they found that, like an antidepressant, exercise induced, in the rat hippocampus, DNA demethylation of the BDNF promoter IV, as well as an increase in the levels of phosphorylated MeCP2 (that, in this form, is released from the *BDNF* gene promoter), thus stimulating BDNF mRNA and protein synthesis [112]. By chromatin immunoprecipitation assay, they also found an increase in the levels of histone H3 (but not H4) acetylation, and a decrease of histone deacetylase 5. In parallel, the levels of CaMKII and CREB increased. Similarly, Ieraci et al. [113] showed that BDNF mRNA (transcripts 1–4, 6, and 7) levels decreased immediately after an acute stress in the hippocampus of mice, then returning to the basal level within 24 h. On the other hand, PA caused an increase in BDNF mRNA and was also able to counteract the stress effect, by inducing an increase in histone H3 acetylation at the level of specific BDNF promoters [113]. Since then, a growing body of studies has shown that PA stimulates an activity-dependent cascade of events, involving phosphorylation and other post-translational modifications of signalling proteins, which arrives at the nucleus, where structural organization and function of the chromatin (which includes, among others, the *BDNF* gene) will be targeted [114].

In conclusion, although all these findings clearly demonstrate a role of PA in regulating the levels of circulating BDNF, the analysis in Table 2 shows that there is no precise exercise protocol that can be favoured in order to obtain a maximal effect on BDNF production and, possibly, on mental health. The authors of these studies/meta-analyses all agree on the need for further research in order to better understand how to use exercise to obtain cognitive improvements.

It is also important to highlight that BDNF circulates in the blood as at least two different pools: BDNF in platelets and platelet-free, plasmatic BDNF. This latter form is probably the only one able to cross the blood–brain barrier (BBB). Thus, the method used to measure the circulating neurotrophin can introduce bias from one study to another. Serum preparations that allow clotting and BDNF release from platelets retrieve a much higher amount of BDNF, in comparison with measurements of BDNF from blood samples containing anti-coagulants [115].

These findings suggest that further experiments based on standardized methods are necessary to understand the real relationship between exercise, BDNF production, and brain health.

### 2.2. microRNAs and Exercise

Recently Zhao et al. [116] obtained, by deep sequencing, a genome-wide identification of miRNAs, the concentration of which is modified in the rat brain, in response to high-intensity intermittent swimming training (HIST), as compared with normal controls (NC). The authors identified a large collection of miRNAs, among which 34 were expressed at significantly different levels in the two conditions; 16 out of these latter species were upregulated, and 18 downregulated in HIST rats [116]. Among the miRNAs that underwent a significant expression modification, some had already been reported by other researchers to be important for brain functions: in particular, the **miR-200 family** had been described to regulate postnatal forebrain neurogenesis [117], differentiation and proliferation of neurons [118], plasticity during neural development [119], and olfactory neurogenesis [120]. Moreover, miR-200b and miR200c seem to have a neuroprotective effect [121]. Actually, most of the predicted targets of PA-controlled miRNAs are genes related to brain/nerve function and already mentioned above, such as *BDNF*, *Igf-1*, *ngf*, and *c-fos*. Some of these genes are also targeted by **miR-483**, another miRNA downregulated in HIST rats [116]. Interestingly, exercise seems to mitigate the effects on cognition of traumatic brain injury and aging by modulating the expression in the hippocampus of **miR-21** [122] and **miR-34a** [123].

In summary, many differentially expressed miRNAs have been evidenced, when comparing the brain of exercising and non-exercising rodents, in a variety of brain areas, including the brain cortex and hippocampus. We have to remember, however, that each miRNA can target a multiplicity of mRNAs, and each mRNA can be targeted by many different miRNAs, thus it is not yet immediately evident how exercise-induced modifications in the miRNA population fit into the general regulation of brain functions by PA.

### 2.3. Genes Involved in Mitochondrial and Lysosomal Biogenesis

Since the 1950s, the decline of mitochondrial oxidative functions has been considered one of the main causes of cell aging [124]. The respiratory complexes (and in particular, the Nicotinamide adenine dinucleotide, NADH, dehydrogenase and the cytochrome C oxidase complexes) decrease with aging in many tissues, including the brain—relying mostly on the oxidative metabolism— that is particularly sensitive to this decline [125,126]. Moreover, mitochondrial DNA (mtDNA) accumulates mutations with age, and this is a further reason for an aberrant functioning of mitochondria [127]. Fission arrest [128] and abnormal donut-shaped mitochondria [129] have been noticed in the prefrontal cortex of aged animals. Mitochondrial alterations of different kinds have been also noticed in a variety of brain pathologies [130,131,132].

On the other hand, PA has been reported to have anti-aging effects and can have a positive effect on mitochondrial biogenesis due to the increase of BDNF levels [133]. Recently, it has been reported that, in old mice, exercise can improve brain cortex mitochondrial function by selectively increasing the activity of complex I, and the levels of the mitochondrial dynamin-related protein 1 (**DRP1**), a large GTPase that controls the final part of mitochondrial fission. This finding suggests that, in the brain of old mice, exercise improves mitochondrial function by inducing a shift in the mitochondrial fission–fusion balance toward fission, even in the absence of modifications in the levels of proteins that regulate metabolism or transport, such as BDNF, HSP60, or phosphorylated mTOR [134].

Autophagy is a physiological process which requires functional lysosomes, and that is involved in recycling proteins as well as in eliminating potentially toxic protein aggregates and dysfunctional organelles [135]. It has been suggested that autophagy is essential in skeletal muscle plasticity and that it is regulated by exercise [135,136,137,138]. Recently, it has been reported that, in the brain cortex, exercise promotes nuclear translocation of the transcription factor EB (**TFEB**), a master factor in lysosomal biogenesis and autophagy [139]. The authors found that activation of TFEB depends on the NAD-dependent deacetylase sirtuin-1 (**SIRT-1**), that deacetylates it at K116, allowing its nuclear translocation. In turn, SIRT-1 is activated by the pathway induced by activation of the AMP-dependent kinase (**AMPK**) [135]. Interestingly, mitophagy (autophagy of mitochondria) declines with age, thus leading to a progressive accumulation of damaged mitochondria [140]. Thus, the autophagy increase, induced by exercise, not only contributes to the elimination of toxic protein aggregates accumulating in the brain, but also produces a specific increase of mitophagy [141].

## 3. Muscle Contraction and Production of Myokines

Skeletal muscle is the most abundant tissue in the body and plays a fundamental role in the maintenance of the correct posture and movement. In addition, it has a central metabolic function, since, in response to post-prandial insulin, picks up glucose from the blood and accumulates it as glycogen. As a consequence, age-related loss of skeletal muscle (known as sarcopenia) not only affects body stability and movement, but might also be a cause of hyperglycaemia. On the other hand, exercise improves glucose uptake in skeletal muscles of patients with type 2 diabetes by activating **GLUT4** translocation to the plasma membrane, partially independent of insulin [142,143].

Different kinds of fibres exist in skeletal muscle, which differs for both metabolic and contractile properties: slow-twitch oxidative (SO) fibres have a high content of mitochondria, and myoglobin, and are more vascularized, fast-twitch glycolytic (FG) fibres have a glycolysis-based metabolism, and finally fast-twitch oxidative glycolytic (FOG) fibres have intermediate properties [144]. Skeletal muscle fibres are also classified according to the myosin heavy chain (MHC) isotypes that they produce: type-I fibres, type-IIA fibres, and type-IIX/IIB fibres, roughly corresponding to SO-, FOG-, and FG-fibres, respectively. Other types of MHC are expressed during embryogenesis or during muscle regeneration [144]. Notably, it seems that also the type of input received from the motor nerve is different for different fibres: type I seems to receive a high amount of inputs at low frequency, while type II seems to receive short inputs at high frequency [145]. Moreover, the contractility properties of muscle fibres do not depend only on the isoforms of contractile proteins expressed, but also on the isotypes of many other proteins, such as those involved in calcium trafficking, and basal metabolism. These differences also depend on epigenetic differences that also influence the transcription rate of the active genes. For example, it has been reported that the mobility of the RNA polymerase II (Pol II) during transcription of the gene encoding PGC-1α differ between fast- and slow-twitch skeletal muscles, thus affecting the gene expression efficiency [146].

Similar to neurons, skeletal muscle cells are post-mitotic, but dynamic, and have the ability to change their structure and physiology in response to long-lasting stimuli, a property called “muscle plasticity” [145]. Thus, for example, fast, fatigable muscles could change to slower, fatigue-resistant ones following chronic electrical stimulation. This remodelling involves an overall change of the structure and metabolism of the fibres, due to modifications of myofibrillar proteins, proteins regulating Ca^2+^ homeostasis, and enzymes involved in glycolysis and in mitochondrial metabolism. All these modifications are time- and intensity-dependent, and imply both transcriptional and post-transcriptional changes of gene expression [145,147]. Adult skeletal muscle can also undergo modifications in response to a more natural way of causing electrical stimulation in the muscles: exercise [148]. One of the factors controlling fibre phenotypes is myoblast determination protein (**MyoD**), a basic helix-loop-helix transcription factor with a critical function in muscle development, that is more highly expressed in fast fibres—in *Myod1*-null mice, indeed, fast fibres shift to a slower phenotype, whereas MyoD overexpression induces the opposite shift [149,150]. A reduction of slow fibres is also observed in calcineurin knock-out mice [151] and in mice overexpressing the calcineurin inhibitor regulator of calcineurin 1 (**RCAN1**) [152]. On the other hand, it has been shown that the nuclear factor of activated T cells (**NFAT**) functions as a repressor of fast properties in slow muscles [153], and is involved in the fast-to-slow phenotype switch induced by aerobic exercise. This effect is due to the NFATs ability to inhibit MyoD action, by binding to its N-terminal transcription activating domain and blocking the recruitment of the histone acetyltransferase **p300** [154]. Interestingly, NFAT is one of the targets of calcineurin-mediated dephosphorylation. It is also worth noting that calcineurin is activated by calcium, and hence by conditions that also trigger muscle contraction.

### 3.1. Muscle Contraction and Gene Regulation

A large body of evidence suggests that muscle contraction per se regulates gene expression and muscle plasticity. Fluctuation in the intracellular [Ca^2+^] is certainly the most important signal during muscle contraction; thus, it is highly probable that the mentioned fibre phenotype modifications and, in general, muscle adaptation to PA are initiated by Ca^2+^. Actually, the molecular basis for contractility depends on the mechanism known as excitation-contraction coupling (ECC), and on the complex interplay between voltage-gated and ligand-gated channels, contractile proteins (such as myosin), calcium-binding buffer proteins (such as calreticulin, parvalbumin, and calsequestrin), calcium-sensor proteins (such as calmodulin and calcineurin), and calcium-dependent ATPases [155].

**Ca^2+^ ions** are also able to regulate glycolysis by making glucose available through glycogen degradation—in muscle cells, glycogen phosphorylase kinase (PhK), the enzyme that phosphorylates and activates the glycogen breaking enzyme phosphorylase (GP), is activated by the calcium/calmodulin (CaM) complex, that constitutes its δ subunit [156,157]. Moreover, CaM can also interact with the muscle-specific isoform of phosphofructokinase (PFK-M), the pacemaker of glycolysis [158]. Ca^2+^ influx into mitochondria also induces an increase in the energy conversion potential, and ATP production [155].

It is also important to highlight that, during muscle contraction, AMP concentration increases, thus activating **AMPK**.

Another important signal due to PA is **hypoxia**; in resting muscle cells, prolyl hydroxylases (**PHDs**) use molecular oxygen to hydroxylate the hypoxia-inducible factor 1α (**HIF-1α**), thus allowing its pVHL (von-Hippel-Lindau) E3 ligase-dependent ubiquitination, and proteasomal degradation [159]. HIF-1α activity is also modulated by the hydroxylation of an asparagine residue (Asn803) by another oxygen-dependent hydroxylase, the factor inhibiting HIF-1 (**FIH-1**); under normoxic conditions, asparagine is hydroxylated, and this modification prevents interaction of HIF-1α with CBP/p300 [160].

In the hypoxic conditions initially induced by exercise, PHDs undergo a decrease of activity, due to shortage of the oxygen substrate, thus hydroxylation of HIF-1α, and hence its ubiquitination and degradation are limited. The stabilized factor translocates to the nucleus, heterodimerizes with aryl hydrocarbon nuclear receptor translocator (ARNT)/HIF-1β, binds to DNA and induces target gene transcription [159]. Genes important for the adaptation of cells to hypoxic conditions and targets of HIFs are, for example, those encoding glucose transporters, glycolytic enzymes, and angiogenic growth factors [161,162].

A further interesting aspect of muscle activity on muscle function depends on **mechanosensing mechanisms**, that depend on forces transmitted to the cells by the extracellular matrix (ECM) or by neighbouring cells during muscle contraction; these forces are simultaneously translated into changes of cytoskeletal dynamics, contributing at the same time to elicit signal transduction pathways [163]. Increasing evidence suggests that a key role in mechanotransduction is played by yes-associated protein (**YAP**), a transcriptional coactivator that can be regulated by ECM stiffness and rigidity, and by cell stretching [163,164]. This protein interacts with different signal transduction pathways, such as the one involving **Wnt/β-catenin** [165], and the one involving **Hippo** [163]. Recently, it has been reported that mechanical stress also activates the c-Jun N-terminal kinase (**JNK**), that then triggers phosphorylation of the transcription factor **SMAD** in a specific linker region. SMAD phosphorylation inhibits its nuclear translocation, thus resulting in a negative regulation of the growth suppressor **myostatin**, and induction of muscle growth [166]. This pathway is activated only by resistance exercise [166]. Interestingly, by using one-legged activity protocols, it was also found that JNK activity increased only in the exercising leg [167]. It is worth noting that global transcriptome analysis, done on muscle biopsies of young men undertaking resistance exercise, revealed that, in the initial exercises, the stress imposed by muscle contraction induced the expression of heat shock proteins (**HSPs**), as well as of muscle damage-, protein turnover-, and inflammation-markers [168]; trained muscles show instead an increase of proteins related to a more oxidative metabolism, and to anti-oxidant functions, as well as of proteins involved in cytoskeletal and ECM structures, and in muscle contraction and growth [168]. Acute resistance exercise also affects the expression of genes encoding components of the ECM, such as matrix metalloproteases, enzymes involved in ECM remodelling [169].

As in the brain, PA-dependent modification of gene expression in muscle mainly depends on epigenetic events. For example, after 60 min of cycling, **HDAC4** and **HDAC5** are exported from the nucleus, thus removing their repressive function [170], and, in general, regular aerobic exercise induces decreased DNA methylation of a number of genes [171,172,173]. Two of the most important epigenetically regulated genes are the above-mentioned **AMPK** and **CaMK** [142,143].

Moreover, exercise induces rapid and transient changes in the muscle miRNAs (also called **myomiRNAs**) [174,175]—for example, after an acute activity bout (cycle ergometer, 60 min, 70% VO_2_ peak), has-miR-1, has-miR-133a, has-miR-133-b, and has-miR-181a increase, while has-miR-9, has-miR-23a, has-miR-23b, and has-miR-31 decrease in the skeletal muscle [175]. Intriguingly, has-miR-1, has-miR-133a, and has-miR-133-b have been instead shown to decrease following an endurance training (cycle ergometer, 60–120 min/section, for 12 weeks, 5 times/week) [176]. As in the case of BDNF, further research is necessary in order to understand the real relationship between PA and miRNA production. Again, the analytic methods used might cause the observed differences, thus, in addition to further studies, it will be necessary to standardize miRNA purification from muscle and blood.

Finally, muscle contraction results in a transient increase of both oxygen and nitrogen reactive species (ROS and NOS, respectively) that, by interacting with redox state-sensing pathways (such as, among others, P-38/MAPK, NFkB, and AMPK), induce cyto-protective, antioxidant responses. Activation of these pathways relies in part on post-translational oxidation of cysteines on critical enzymes/regulatory proteins by glutathionylation, that is by reversibly adding glutathione to their thiol groups; in addition to stimulating protective cell responses, this modification probably prevents further irreversible oxidation of cysteines [177,178].

### 3.2. Release of Myokines and Metabolites by Contracting Muscles

As a whole, the data reported indicate that PA has several effects on the nervous system—it acts as an antidepressant and an anxiolytic, and can improve mood, self-esteem, and cognition. The benefits induced by PA on the brain (as well as in other organs, such as the heart) are in part mediated by peptides (myokines) and metabolites released into the blood by the endocrine activity of contracting muscles (Figure 1) [25,179,180,181,182].

#### 3.2.1. BDNF and Cathepsin-B (CTSB)

Contracting muscles release BDNF, that seems to be involved in autocrine signalling to the muscle itself [182,183,184]. In addition, BDNF probably serves as a retrograde signal to the motor neurons of the spinal cord.

It is also possible that muscle-derived BDNF has an effect on the brain, as intact BDNF was reported to cross the blood–brain barrier (BBB) in both directions by a high-capacity, saturable transport system [185].

In response to exercise, muscles also release into the plasma high levels of cathepsin-B (CTSB), an abundant, calcium-dependent cysteine protease of the calpain family, produced in all human tissues. Enzymatically active CTSB is secreted through exocytosis and can degrade components of the ECM in both physiological and pathological conditions [186,187]. Although the mechanism of action of CTSB in the brain is still a matter of debate, it was acknowledged that, after exercise-dependent release from muscles, it can cross the BBB and promote BDNF expression in the hippocampus, neurogenesis, and promote the improvement of spatial memory abilities [188]. It has been reported, for example, that in CTSB knockout mice, running did not have any effect on hippocampal neurogenesis and spatial memory [188]. Similarly, in humans, changes in CTSB levels correlate with hippocampus-dependent memory functions [188].

Intriguingly, it was recently reported that resting serum levels of both BDNF and CTSB were significantly lower in long-term trained middle-aged men in comparison with sedentary controls, even if trained men showed a significant improvement in memory, based on the Free and Cued Immediate Recall tests [107]. Thus, it seems that both BDNF and CTSB molecules increase immediately after exercise, but then decrease to levels lower than in untrained individuals, showing an inverse correlation to the intensity/duration of exercise ([107]; Table 2). It is tempting to speculate that, as proposed years ago by Ji et al. [189], and as discussed by De La Rosa et al. [107] for BDNF and CTSB, most of the regulatory molecules produced in response to PA behave in a hormetic manner: in other words, their concentration should increase at the beginning of the activities, when they play an immediate role in repair processes, at the sites of the traumatic injuries, where oxidative stress is initially induced by exercise. Then, in well-trained individuals, given the better adaptation to stress, the levels of these molecules could/should decrease. Thus, their concentrations over time, if put in a graph, should give rise to a curve with the shape of an upside-down “U”. Such behaviour might also represent one of the variability sources in results found in different studies—analyses performed at different time intervals during and after the exercise might give rise to very different evaluations and interpretations.

#### 3.2.2. FGF21 and Irisin/FNDC5

FGF21 is primarily produced by the liver, but also by skeletal muscles [179,190]—it is a critical regulator of nutrient homeostasis [191]—in response to PA, it improves thermogenesis in adipose tissue and skeletal muscle, and even induces differentiation of brown adipocytes [192]. FGF21 also crosses the BBB [193] and, in association with the co-receptor βKlotho [194], binds to its receptors in the hypothalamus, where it modulates sympathetic input to brown adipose tissue, circadian rhythms, and neuroprotection [182]. Recently, it has been shown that, although all exercise types induce an increase of FGF21, the increase is greater after resistance training than after high-intensity interval (HIIT) sessions [195].

Irisin, the proteolytic cleaved extracellular part of fibronectin type III domain-containing protein 5 (FNDC5), is a myokine the expression of which depends on PGC-1α [196], and that is positively regulated by muscle contraction [197]. Like FGF21, upon its release into the systemic circulation, irisin may contribute to the browning of white adipose tissue [196]. FNDC5 has been detected in different areas of the brain, where it seems to associate with neural differentiation; moreover, irisin can cross the BBB [198], and increased levels of circulating irisin correlate with increased levels of BDNF in the mouse hippocampus [197].

#### 3.2.3. Cytokines Released by Muscles

Contracting muscles also release cytokines, such as **IL-6**, **IL-8**, and **IL-15**. As interleukin passage across the BBB has been reported [199], these molecules are putatively able to act on the brain too. Their effects on the brain are, however, still debated. For example, both neurodegenerative and neuroprotective properties have been attributed to IL-6 [200]; interestingly, it seems that these different effects depend on the receptors engaged, and the specific signalling pathway triggered: (1) The anti-inflammatory pathway (the classical one) involves the membrane-bound IL-6 receptor (IL-6R), expressed for example on microglia, and (2) the pro-inflammatory one (also called the trans-signalling pathway), mediates neurodegeneration in mice, and depends on a soluble form of IL-6R, able to stimulate a response on distal cells [200]. Similarly, IL-8 seems to have both neurogenic and neurotoxic effects [201]. IL-15 receptors are expressed by both glial cells and neurons, with developmental and regional differences. A neuroprotective role of IL-15 is suggested by the increased motor neuron death in knockout mice lacking the IL-15 receptor α (IL15Rα), and by the ability of IL-15 treatment to ameliorate the symptoms of the experimental autoimmune encephalomyelitis (EAE). On the other hand, increased blood levels of IL-15 have been observed in inflammation of several origins [202]. In summary, as in the case of BDNF and CTSB, the levels of the muscle-derived interleukins probably have a hormetic behaviour, and their changes depend on the general adaptation to stress.

#### 3.2.4. Lactate

It is now widely acknowledged that **lactate**, produced in large amounts during anaerobic exercise, shuttles among cells and, inside the cells, among organelles, through specific monocarboxylate carriers (MCTs); interestingly, lactate behaves as a fuel for many cells, including neurons, in conditions of oxygen shortage [203]. Moreover, the hydroxycarboxylic acid receptor 1 (HCAR1), a G protein-coupled lactate receptor, is highly enriched in the endothelial- as well as in the pericyte-like-cells of the intracerebral microvessels. Activation of HCAR1 enhances production of the cerebral vascular endothelial growth factor A (VEGFA) and cerebral angiogenesis [204,205]. More recently, it was also found that, by signalling through HCAR1, lactate can activate responses that involve both α and βγ subunits of HCAR1 and is synergic with the activity of other receptors, such as adenosine A1, GABAB, and α2-adrenergic receptors. As a consequence, not only neurons can use lactate as a substrate during exercise but, in addition, neuronal activity might be finely tuned by this molecule [203,206]. These findings highlight the important role that lactate can play in the PA-dependent muscle–brain crosstalk.

#### 3.2.5. Extracellular Vesicles (EVs)

In the last two decades, many laboratories have demonstrated that cells can communicate at long distances by releasing EVs (mainly exosomes and/or small membrane vesicles/ectosomes) that contain many species of proteins, nucleic acids, lipids, and metabolites. Since they are membrane-bound, EVs can also fuse with the plasma membranes of other cells, thus delivering their content into them and inducing epigenetic modification of the recipient cell functions [207,208]. Central protagonists of EV-mediated trafficking are different species of RNA, and especially miRNAs. Although many obstacles are still encountered in the identification and purification of EV-carried circulating miRNAs [209], many laboratories have reported that PA induces a significant modification of many miRNAs. Among these latter species, some (for example, miR-21 and miR-132) have a role in brain functions as critical as regulation of synaptic plasticity, memory formation, and neuronal survival [82].

It is thus possible that one of the ways through which PA and muscle activity influence brain function is by delivering into the blood different species of regulatory molecules that are protected during the trip to other tissues (and to the brain, in particular) because they are packaged into EVs, and these membrane-bound vehicles might finally deliver their cargoes to the brain across the brain capillary endothelial cells. Interestingly, indeed, exercise stimulates the release of exosomes and small vesicles into circulation [210].

## 4. A Few Examples of Exercise Effects on Neurodegeneration: Studies on Alzheimer’s Disease, Parkinson’s Disease, Huntington’s Disease, and Multiple Sclerosis

The evidence that regular exercise can help to prevent and even treat neurological disorders has become stronger in recent years. At the same time, a lot of research is focusing on the mechanisms underlying the ability of PA to improve the symptomatology of neurodegenerative diseases, in the attempt to find out the best protocols to be applied to the patients.

### 4.1. Alzheimer’s Disease (AD)

PA improves cognition in a mouse model of Alzheimer’s disease (AD), stimulating neurogenesis and the simultaneous increase of both BDNF and FNDC5 [211]. For example, intracerebroventricular- or tail vein-injection of FNDC5 allowed the recovery of memory impairments and synaptic plasticity in a mouse model of AD [212]. Since PA, as already discussed in Section 3, stimulates irisin release from muscle, it is possible that the beneficial role of PA is, at least in part, due to this myokine [213]. Interestingly, the effects of irisin could be also attributed to a lower release of inflammatory cytokines by astrocytes—it was shown, that irisin has protective effects on cultures of hippocampal neurons treated with Aβ peptide, only when co-administered with astrocyte-conditioned medium [214].

In the hippocampus, PA effects include: (i) enhancement of **c-Fos-** and **Wnt3-** and inhibition of glycogen synthase kinase-3β (***GSK-3β***)-gene expression; (ii) an increase of glial fibrillary acidic protein (**GFAP**) and a decrease of the **S100B** protein levels, in astrocytes; (iii) an increase of the blood–brain barrier integrity; (iv) an increase of BDNF and tropomyosin receptor kinase B; (v) enhancement of glycogen levels; and (vi) normalization of MCT2 expression [215].

Another AD progression-slowing factor, known to be produced during physical activity and able to cross BBB is the insulin-like growth factor 1 (IGF-1) [216]. This factor acts by activating the expression of BDNF. If antibodies against its receptor are used, the PA-induced increase of BDNF mRNA, protein, and precursor does not occur anymore [217].

Moreover, as already discussed (Section 3), both lactate and BDNF produced during physical exercise seems to have stimulating effects on learning and memory processes [205]. As mentioned, CTSB also crosses BBB, and should be able to stimulate hippocampal neurogenesis, and to improve learning and memory, however, AD patients have high levels of this enzyme in the blood, thus, the real role of CTSB in AD remains controversial [205].

Some miRNAs, such as miR-124 and miR-134, have been also suggested to be involved in memory formation and maintenance [218,219]. The relationship between the role of these molecules and BDNF in AD is, however, still debated [220]. Interestingly, in a mouse model of AD, **miR-34a** is upregulated [221]; it has been hypothesized that swimming training, by inhibiting miR-34a expression, might attenuate age-related autophagy dysfunction and abnormal mitochondrial dynamics, thus delaying both physiological brain aging and AD [123].

An altered metabolic process in AD is that involving demolition of the L-tryptophan, which leads to the formation of kynurenine (**KYN**); catabolism of this latter molecule generates, in turn, neurotoxic metabolites related to AD pathogenesis. KYN is able to cross freely the BBB and, in AD patients, it is found in excess both in the plasma and in the brain. PA might be protective for neurons because it stimulates the formation of an aminotransferase (**KAT**) in the muscle, KAT then catalyses the peripheral transformation of KYN into kynurenic acid (**KYNA**), and is less harmful because it is unable to cross the BBB [182,205].

Recently, it has been also reported that 4 weeks of exercise can revert the induction of gene encoding proteins involved in inflammation and apoptosis in the hypothalamus in a mouse model of AD. After 6 weeks, an improvement in glucose metabolism was also observed, and after 8 weeks there was an evident reduction of apoptosis in some populations of hypothalamic neurons [222]. Finally, it has been suggested that the benefits noticed in early AD patients following aerobic exercise are due to the exercise-dependent enhancement of the cardiorespiratory fitness, which is in turn associated with improved memory performance and reduced hippocampal atrophy [223].

### 4.2. Parkinson’s Disease (PD)

Parkinson’s disease (PD) is the second most common neurodegenerative disorder and involves a massive degeneration of the dopaminergic neurons in the substantia nigra, in the midbrain [224]. Although the priming cause is still unknown, both genetic and environmental cues could play a role. Some of the familial cases show mutations in the gene encoding α-synuclein, a protein mainly found in the presynaptic terminals; the mutated protein is prone to aggregation and tends to form the so-called Lewi bodies, which contribute to the degeneration of neurons [225,226].

At present, pharmacological therapies able to remarkably modify or delay the disease progression, are still lacking. Thus, alternative approaches not entirely based on pharmacotherapy, and able to slow down the dopaminergic neuron degeneration, are needed. Also, in the case of PD, it has been reported that association of the pharmacological therapy with exercise can help in managing the physical and cognitive decline typically associated with PD [227]. Several studies investigated the effects of various types of exercise on both motor- and non-motor-features of PD and reported positive results: 19 systematic reviews and meta-analyses, from 2005 to 2017, were published from which an increased interest in non-pharmacologic therapies is evident [228,229].

In PD animal models, exercise induces neuroprotective effects through the expression of some brain neurotrophic factors, including BDNF and glial-derived neurotrophic factor (GDNF) [230,231]. In particular, it was demonstrated that the promoter IV of *BDNF* gene shows a reduced CpG methylation in rat, after regular enrolment in physical exercise [112]. Moreover, free-wheel running (from 1.6 to 7 km/day) could improve histone H3 phospho/acetylation and c-Fos induction in dentate granule neurons [232]. These observations suggest that the positive effects of PA depend on epigenetic regulation of genes encoding neurotrophins.

Other exercise effects in PD animal models include enhanced cell proliferation and migration of neural progenitors, and an overthrow of age-related deterioration in substantia nigra vascularization, that seems to be mediated by VEGF expression [233]. Moreover, a study on PD mice models highlighted that, after a 6-weeks treadmill training exercise, a nigrostriatal Nrf2-ARE (antioxidant response element)-dependent signalling pathway was activated, which was protective against the development of parkinsonism [234].

Treadmill running also enhanced coordination and motor balance by preventing loss of Purkinje cells in the rat cerebellum. Moreover, repression of PD-induced GFAP-positive reactive astrocytes and Iba-1-positive microglia was found, showing that PA can help in suppressing astrogliosis and microglia activation. These cellular effects were accompanied by a decreased expression of the pro-apoptotic protein Bax, and enhanced expression of the anti-apoptotic protein Bcl-2 [235].

In humans, the effects of PA have been studied on the basis of correlations found among acute effects of exercise on specific clinical variables (as emerging, for example, from the PD Questionnaire-39 on quality of life) and the amplitude of low frequency fluctuations (ALFF) that may reflect the functions of the brain before and after a single bout of exercise. The results of these analyses showed, for example, an increase of ALFF signals within the right ventromedial prefrontal cortex (PFC) and the left ventrolateral PFC, as well as a bilateral increase in the substantia nigra [236].

Another study demonstrated that 4 weeks of aerobic exercise elicited a long-lasting improvement on both motor and non-motor functions of PD patients. The principal result of this study was an increase of BDNF signalling through its TrkB receptor in the patient’s lymphocytes [237]. Similar results had been also reported by Wang et al. [238], who found that repetitive transcranial magnetic stimulation enhanced BDNF-TrkB signalling in both brain and lymphocytes [238]. It is thus possible that BDNF-TrkB signalling in lymphocytes can be indicative of what happens in the cortical TrkB signalling.

On the basis of these studies, we can conclude that PA can give PD-specific clinical benefits, but only if repeated habitually over time (i.e., exercise training) [239].

### 4.3. Huntington’s Disease (HD)

HD is a fatal genetic disorder, due to an autosomal dominant mutation that determines the expansion of poly-glutamine repeats in the huntingtin (HTT) coding region [240]. Clinical features of HD include significant motor defects together with non-motor changes, like cognitive, psychological, and behavioural disabilities, that may progressively get worse before diagnosis, and that results in limitations of daily activities [241]. Physical therapy and exercise interventions were integrated into the treatment decades ago, in order to maintain patient’s independence in daily life activities, while attenuating the damages in the motor function. It is indeed known that a passive lifestyle might lead to an earlier HD onset; while, as in other neurodegenerative diseases, exercise exerts a positive effect [242,243]. Recent studies have focused on both resistance and endurance exercise training modalities, based on the suggestion that both could be of help in HD patients. All the results showed a significant increase in grey matter volume and significant improvements in verbal learning and memory, after long-training exercise [243,244,245,246,247,248].

Interestingly, it was highlighted that voluntary exercise in a rat model of HD induces DNA hypomethylation at specific CpG sites, located within an Sp1/Sp3 transcription factor recognition element of the *vegfA* gene promoter. In parallel, a significant reduction of the mRNA encoding DNA methyltransferase 3b (DNMT3B) in the hippocampus of exercised rats was also found [249].

### 4.4. Multiple Sclerosis (MS)

Patients with Multiple Sclerosis (MS) who perform regular physical activity have a better quality of life with less fatigue and less depression than those who are sedentary [250].

A pilot study with relapsing-remitting MS patients demonstrated that exercise may also attenuate inflammation and neurodegeneration by an increase of **erythropoietin** [251].

Mulero et al. [252] analysed gene expression in MS patients who improved their fatigue status after an aerobic exercise program and compared them with healthy controls (HC). It revealed that in patients before exercise, genes that respond to interferon were more active than in the HC. On the other hand, after training, a decrease in the expression of a group of interferon-related genes was evidenced at the transcriptomic level [252]. These results are encouraging because the expression of genes activated in response to interferon also correlates with the increase in fatigue [252]. Exercise also induced a reduction of the levels of the IL-6 receptor, that went back to normal values [252]. Moreover, in the hippocampus of an animal model of MS, both high- and low-intensity training programs induced an increase of the mRNAs encoding three important neurotrophins: BDNF, the glial-derived neurotrophic factor (GDNF), and the nerve growth factor (NGF) [253].

In addition to the PA-dependent increase of BDNF, VEGF, and IGF-1, in the context of MS, a specific increase in the expression of tight junction proteins, critical for the reestablishment of the BBB function, was also evidenced [254]. Moreover, using a mouse model of MS with overexpressed ATP-binding cassette transporter 1 (**ABCA1**), Houdebine and colleagues [255] demonstrated a PA-dependent normalization of ABCA1 mRNA levels both in the brain and the cerebellum, with an improvement of myelin status.

Actually, it has been also found that different training protocols act differently on gene expression; for example, while IGF1-R expression level decreases in the brain of MS mice subjected to forced-swimming protocol, IGF1-R mRNA level increases in the cerebellum of MS mice of a running group. In parallel, a different pattern of myelin gene stimulation was also observed—in the mice that had performed running exercise, a smaller decrease of myelin was found in the brain, whereas swimming induced greater benefits in the cerebellum [255].

In summary, these few examples of PA benefits in different neurodegenerative diseases reinforce the idea of a neuroprotective effect of exercise. Exercise increases expression of genes involved in enzymatic antioxidant responses, improves cognitive functions and memory, and can counteract the progression of diseases, or at least help patients to better perform daily life activities.

There are probably multiple cellular and molecular pathways involved and act in synergy. Moreover, specific differences in the responses of individual patients can be expected depending on genetic and epigenetic variability as well as even slight differences in the grade of the pathology.

Finally, the protocols used in different studies are highly heterogeneous and to set ideal exercises for the different neurodegenerative pathologies is at the moment impossible. Further research is still necessary, and, as already noticed above, standardized methods for analysing the results and the biomarkers are compelling.

In spite of the mentioned uncertainties and variability, the current results are of real interest and encouraging. Moreover, the understanding that many biochemical pathways are involved has been stimulating a lot of new studies, aimed at finding out the best combinations of exercise and drugs to slow down the pathology while improving the life quality of the patients.

## 5. Exercise-Dependent Production of Dopamine, Endocannabinoids, and Opioids: Effects on Mood, Analgesia, and Happiness

In addition to an improvement of body fitness and learning and memory skills, it is well documented that PA can induce changes in the mental status, reducing anxiety and producing a general sense of wellbeing. Moreover, it can induce analgesia. The precise mechanisms involved are not yet completely understood but a few molecules, probably acting in synergy, have been identified and are currently studied as possible mediators of these further effects of PA.

### 5.1. Dopamine

Dopamine (DA) producing neurons are present in distinct areas of the cerebral cortex, but are mostly concentrated in the ventral midbrain, where they are arranged in different nuclei. The two main groups constitute the pars compacta of the substantia nigra (SNc), and the ventral tegmental area (VTA). The latter neurons send projections to the nucleus accumbens of the ventromedial striatum, but also to the limbic system and the prefrontal cortex, being mostly involved in the regulation of emotional, reward-related, and cognitive functions. Dopaminergic neurons of the SNc, which regulate mainly motor function, form the nigrostriatal pathway, innervating neurons located in the caudate nucleus and in the dorsolateral striatum [256,257].

This subdivision is probably an oversimplification because different subgroups of DA neurons have recently been described in human and murine midbrain, which show distinct gene expression profiles [258,259]. Even though it is not yet known whether these DA neurons have specific roles, in some instances it was demonstrated that they have unique projection patterns, connecting them to distinctive areas such as the nucleus accumbens and amygdala [260]. Using single-cell RNA sequencing, and PITX3 protein and tyrosine hydroxylase (TH) as markers for DA neurons, Tiklovà et al. [261] identified seven different populations of neurons in the mouse developing midbrain, that could be distinguished thanks to the differential expression of other genes [261].

DA neurons appear to form a brain network regulating the motivational behaviour of animals, allowing them to learn the difference between useful and harmful things, and consequently to choose proper actions [262]. DA also seems to be necessary for performing motivated actions to achieve goals, as demonstrated by the unsuitable behaviour of dopamine deficient mice [263]. In the mammalian central nervous system, DA controls many processes [262,264], such as feeding and locomotion [265]; it is also involved in the mechanisms of cognition and ‘adaptive’ memory formation, influencing the hippocampal long term potentiation (LTP) [266], and upregulates BDNF in the prefrontal cortex [267]. DA most probably interacts with other neurotransmitters and neuromodulators and, for example, it has been recently demonstrated that midbrain mice DA neurons also release IGF-1 that modulates DA release and concentration as well as neuronal firing [268].

As mentioned, a lot of different evidence demonstrates that the mammalian brain is capable of changing its functional and structural characteristics to adapt to the ever-changing surrounding world. This is achieved by learning and acquiring skills, thus improving cognitive functions. Neuroplasticity is orchestrated by several neurotransmitters and neurotrophins, and many cues indicate that exercise has an important role in its regulation [269]. In particular, DA regulates emotion and reward-related brain functions, and many authors have postulated that the positive properties of PA may be due to its ability to increase DA concentration [270,271,272]. Interestingly, PA increases the concentration of the same neurotransmitters, including DA, also activated by some drugs and alcohol [273], and this could be the reason why it improves mood in humans [274,275].

Moreover, PA, and specifically voluntary exercise, creates a sharp increase in DA concentration, especially in the nigrostriatal pathway, and has a strong positive effect in overcoming aversion. Being a molecule involved in the regulation of movement, emotions, and learning, DA could be a key component in the mechanism. Nevertheless, even though many proofs about DA’s involvement in the beneficial effects of exercise have been accumulating, to date a clear explanation of the underlying mechanism is still missing [276].

One interesting aspect of DA function is that it appears as one of the factors that distinguish physically active organisms from inactive ones, influencing the locomotory activity and even the tendency of the individuals to engage in PA [277]. Voluntary exercise is genetically controlled and depends on different neuromodulators, including DA itself. Given the enhancing effects of PA on DA production and release in the brain, it can be hypothesized that an auto-sustaining circuit exists by which DA and PA positively interact—the more DA an individual animal produces, the more it is prone to live actively, and the more DA will be consequently released in this feed-forward system [278].

Even though the mechanisms by which exercise, through dopamine, creates positive effects on brain functions are yet to be elucidated, a few hypotheses have been proposed. For example, it has been demonstrated that voluntary wheel running (VWR) activates latero-dorsal tegmental (LDT) and lateral hypothalamic area (LHA) murine neurons and these, in turn, could be responsible for the activation of the DA neurons of the lateral ventral tegmental area (lVTA) [279].

DA increase in the brain can derive from a higher activity of the tyrosine hydroxylase (TH) enzyme, most probably due to a rise in calcium concentration. Enhancement of the enzyme activity depends indeed on its phosphorylation by CaMKII, the activity of which is regulated by calcium [280,281]. Actually, wheel running in rodents causes a doubling of the TH mRNA level in the VTA [282], and an increase also in the substantia nigra [283], and in the locus coeruleus [284]. A chronic exercise-dependent high level of DA, but not of other neurotransmitters (such as noradrenalin, serotonin, or glutamate), in the rat medial prefrontal cortex (mPFC), was found by Chen et al. [285], and the effect could be reduced by a glucocorticoid receptor inhibitor—the authors suggest that the local DA increase is due to the high level of cortisol, induced by PA in mPFC [285].

In summary, PA may cause an increase in serum calcium levels, and calcium can stimulate dopamine synthesis in the brain by stimulating the activity of the CaMKII, and the consequent activation of the TH enzyme by phosphorylation. In particular, it has been shown that mice forced to physical activity have a DA level sharply higher in the neostriatum and nucleus accumbens, and that a similar effect, i.e., a specific increase of DA level in these brain regions, can be obtained by intracerebroventricular injection of calcium chloride. Moreover, following physical activity, a significant amount of TH and CaM was found in mouse neostriatum and nucleus accumbens, and in human, was found in the caudate nucleus and putamen. A possible mechanism leading to calcium increase in the brain could be the release of lactate following exercise. This may induce, in turn, an increase of blood acidity that could activate parathyroid hormone, or directly increase calcium concentration by favouring bone resorption [286].

On the other hand, PA-dependent DA increase might also be a consequence of a decrease in the activity of catabolic enzymes, such as the mitochondrial monoamine oxidase (MAO) and the catechol-O-methyltransferase (COMT) [287]. In a study aimed at associating genetic background to happiness, Chen et al. [288] found that women bearing the low expression MAO-A alleles are statistically happier than those bearing the high expressed variant. Surprisingly, no difference in happiness was found when comparing men bearing the two different type of alleles [288]. Similar results have been reported regarding the COMT gene—women bearing a particular allele, containing the COMT Val158Met polymorphism, and presenting as a consequence a higher DA concentration show an emotionally healthier behaviour [289].

### 5.2. Opioids, Endocannabinoids, Analgesia, and the “Runner’s High”

The endogenous opioid system includes different peptides (i.e., endorphins, enkephalins, and dynorphins) that derive from larger precursors and bind to G protein-coupled receptors. Three main receptors (μ, κ, and δ) mediate analgesic effects of these molecules [30]. Several studies have demonstrated PA-dependent increase of circulating opioids, and in particular of β-endorphin, in relationship with the intensity of exercise, and this β-endorphin increase correlates with analgesic effects both in humans and in rodents. Many studies, however, suggest that opioids are not the only molecules involved in analgesia induced by exercise [30]. For example, activation by exercise of the mesolimbic system in rodents has been also related to analgesic effects [290].

The endocannabinoid system (ECS) includes two G protein-coupled cannabinoid receptors (CB1 and CB2), widely expressed all over the body, and their endogenous ligands, the most well-studied of which are two derivatives of the arachidonic acid: N-arachidonoylethanolamine (AEA, also known as anandamide) and 2-arachidonoylglycerol (2-AG). ECS also includes the enzymes necessary for synthesizing and degrading the ligands [291]. In addition to CB1 and CB2 receptors, 2-AG and AEA can bind to the vanilloid receptor (TRPV1); moreover, AEA also functions as an agonist of some subtypes of the peroxisome proliferator-activated receptor (PPAR) family of nuclear receptors [292]. ECS is critically involved in the modulation of several aspects of metabolism, and, in the hypothalamus, endocannabinoids signalling seems to function in maintaining appetite, in contrast with leptin. In particular, CB1 is probably involved in reward circuits related to food (i.e., it is responsible for the hedonic aspect of eating) [292]. An expected consequence of these ECS functions is increased production of endocannabinoids in response to exercise that induces higher energy utilization. A variety of studies have indeed shown PA-dependent increase of circulating endocannabinoids, even if the results significantly differ from one study to another. It seems, for example, that the relationship between the increase of AEA and the exercise intensity, as in the case of other already mentioned molecules, is described by an “upside-down U”-shaped curve [292,293]. On the other hand, 2-AG was found significantly elevated in response to short and intense bursts of activity. It is thus possible that different endocannabinoids (or different mixes of them) are secreted in response to different types, intensities, and durations of exercise. Moreover, “preferred” exercises significantly activate ECS, and this response may also contribute to the effects on the mood [294].

Interestingly, it was also reported that hypoxia potentiates ECS activation, and it was suggested that the muscles can be the main source of the exercise-induced increase of circulating endocannabinoids, that then can cross the BBB [292]. Overall, the levels of circulating endocannabinoids are inversely related to anxiety and depression, and positively related to BDNF concentration and, thus, to the beneficial effects on mood and to a sense of vigour and wellbeing. However, 2-AG and/or AEA levels can be higher in patients with schizophrenia or other cognitive disorders, such as borderline personality disorder [292]. Moreover, these observations are consistent with the evidence that the use of cannabinoid drugs increases the risk of developing psychotic disorders, probably also in relation to alteration of the dopamine signalling [295]. In summary, it is highly probable that these molecules can also have hormetic behaviour (see Section 3.2.2).

Since the 1960s, it was known that long-running could cause what was called the “runner’s high”, a sudden sense of euphoria and wellbeing, accompanied by analgesia. For a long time, exercise-dependent production of endorphins was considered responsible for at least the analgesic component of the runner’s high. More recently, as mentioned, the involvement of both opioids and endocannabinoids in this aspect of the response to PA has been consistently reported [30], and, in addition, it was found that cannabinoid-agonists can enhance the release of endogenous opioids in the brain [295]. We can thus infer that the two systems act in synergy in the anti-nociceptive effects of exercise. It has been also reported that, at the molecular level, a mediator of endocannabinoid action in response to exercise is AMPK [296].

On the other hand, most other aspects of the runner’s high seem to depend more directly on the endocannabinoid receptors, in mice [297], even if it is not so easy to evaluate euphoria in mice.

It was also suggested that mood improvement could relate to PA-dependent increase of the levels of neurosteroids, and in particular of dehydroepiandrosterone (DHEA) [298], a molecule with a variety of effects on different neurotransmitter receptors, such as the GABA_A_ receptor, and the NMDA as well as the AMPA receptors for glutamate. DHEA can also bind to nuclear receptors, can contribute to regulating the mitochondrial function in response to stress, and, through activation of G-protein coupled receptors of the plasma membrane, can increase transcription of miR-21, at least in a cell line of hepatocytes [299].

## 6. Conclusions and Perspectives

In conclusion, habitual exercise has a variety of positive effects on the human body, from regulating cardiorespiratory and cardiovascular fitness, to improving glycaemia and insulin response. In addition, as discussed, it is a way of maintaining not only a healthy body, but also a healthy mind, at any age. In particular, it can represent a non-pharmacological (and sometimes enjoyable) strategy to delay the effects of both physiological ageing and pathological neurodegeneration on brain health. However, although exercise prescriptions (including frequency, intensity, type, and time) were given, for example, for individuals with hypertension ([20], Table 1 in [23]), we cannot yet refer to specific exercise prescriptions for maximizing the positive effects of PA on cognition [23]; the protocols used in the experiments reported in this review, as well as the subjects and the markers studied (Table 1 and Table 2) are indeed quite different, many informative studies relied on rodents, and not yet on humans. Further studies are thus necessary to evaluate more precisely how the factors which influence brain functioning change in response to the type, intensity, and timing of exercise. Further studies are also required to understand the interplay among the many molecules the levels of which change during/after exercise, even in opposite directions. PA induces, indeed, a variety of cellular and molecular effects, both in the periphery and in the brain. As we have seen, every molecule/group of molecules probably affects different aspects of brain function, but their synergic effects contribute to brain health as a whole. Among all these factors a key role seems to be played by BDNF—as a PA effect, this latter molecule is produced in the periphery and can also cross the BBB. In addition, some BDNF is directly produced in the brain due to the effect of other molecules, some of which are similarly released in the periphery, in a PA-dependent manner, and then cross the BBB, where they affect the function of resident proteins either at the transcriptional or the post-transcriptional level.

Notably, all these effects also depend on the physical pre-exercise conditions of each person.

In this context, an additional issue arises from the actual difficulties of old people and patients with neurodegeneration to perform voluntary exercise. Interestingly, a recent paper reported that neuromuscular electrical stimulation (NMES) can increase BDNF and lactate serum concentration even more than voluntary exercise, and might thus represent a solution for individuals who cannot engage in high-intensity exercise or are even unable to perform any exercise at all [300].

It should also be considered that a significant percentage of people with a sedentary, computer-dependent lifestyle consider physical activity not only hard but also boring, and thus lacks motivation to exercise. From this point of view, a challenging proposal was made in a recent paper, in which the authors reported that a virtual reality-based exercise can be of help for people who cannot and/or do not like to move [301]. Namely, they refer to a particular type of dual-task video-games (exergames) that require, on behalf of the player, also a certain degree of movement, with variable physical components. The authors suggest that exergames can be also useful for children with impaired motor functions, for people who undergo rehabilitation, and for the elders [301]. Perhaps, the possibility to perform PA in the context of a virtual game can help also in the case of children and adolescents with intellectual disabilities (ID); it has been reported that children with moderate to severe ID also suffer from low physical fitness [302].

Notably, we are now aware that pharmacological therapies should be ideally shaped on individual patients because of genetic and epigenetic differences affecting responses to the drugs. When talking of physical activity, tailoring prescriptions to individuals is even more difficult since the ability to perform exercise as well as the exercise outcomes likely depend on a wider set of genes (and their epigenetic setting). For example, single nucleotide polymorphisms have been observed in a number of genes that encode proteins involved in PA/fitness relationship, such as, for example, the genes encoding BDNF and the e4 allele of apolipoprotein apoE [56], or the genes encoding muscle proteins, such as actinin [303]. These considerations become much more stringent when we focus on the nervous system—every brain is unique because of genetic and epigenetic peculiarities that accumulate throughout our lives, as an effect of learning and experiences that sculpt our mind [304].

Now, although these concepts are clear at the theoretical level, practical applications are still in their infancy, even if rapidly progressing. In the future, animal models will certainly be helpful to study correlations among specific genes and exercise outcomes. On the other hand, further studies on humans will be helpful, provided that more homogeneous interventions and standardized measurement methods are used to evaluate exercise-dependent modifications of key parameters.

Finally, as life expectancy is increasing all over the world, it is of the utmost importance for all of us to maintain independence in the daily life activities and a sense of wellbeing as long as possible. Since PA can clearly contribute in ameliorating physical fitness as well as the mental status, it should be a social and political task to promote the conditions that allow the realization of physical exercise programs for the entire population, and especially for the elders and for children. In particular, we suggest that both healthy people and patients are encouraged by physicians to perform physical activity, underlining the higher impact and efficacy of moderate and regular exercise, in comparison with acute and heavy bouts of activity.

## Figures and Tables

**Table 1:** Effects of physical activity (PA) on learning and memory in children and adolescents. In the first three rows single studies are reported, while the second three rows refer to reviews/meta-analyses. In the “Conclusions” column, the main results of the analyses, as well as a few comments on them, are given.

Protocol/Aims [Ref]	Subjects/Studies Included	Methods of Analysis	Conclusions
Analysis based on a randomized controlled trial (Ballabeina Study: [65]) aimed at evidencing any relationship between aerobic fitness/motor skills and working memory and attention in pre-school children [59]	245 ethnically diverse pre-school children (49% girl, mean age 5.2 years) were analysed at the beginning of the activity and 9 months later.	Physical tests: 1. Aerobic fitness, assessed according to the 20 m shuttle run [66], 2. Agility, assessed by an obstacle course, 3. Dynamic balance on a beam. In order to evaluate spatial memory and attention, each child was tested individually by focused tests.	**Higher baseline aerobic fitness and motor skills were related to higher levels of working memory and attention.** A further improvement of these latter abilities was noticed in the following 9 months.
The aim of the study was to ascertain whether very low-intensity exercise (i.e., walking), practiced during foreign-language (Polish) vocabulary encoding, improves subsequent recall, in comparison with encoding during physical rest [62]	49 right-handed, monolingual, Germans, healthy subjects (aged 18–30 years). Criteria of exclusion: a history of psychiatric or neurological disorders, smoking, obesity, and any knowledge of Polish or other Slavic languages.	In the first session, participants learned 40 Polish words while walking on the motor-driven treadmill, at their previously determined preferred rate. In the second session, the participants learned a further group of 40 words, while sitting in a chair. Each session lasted 30 min. The order of sessions was different for different subjects, in a balanced way, and the experiments were repeated twice.	In both experiments, participants’ **performance was better when they exercised during learning** compared to learning when sedentary. Serum BDNF levels and salivary cortisol concentration were also measured: serum BDNF was unrelated to memory performance; on the other hand, a positive correlation between the salivary cortisol and the number of correctly recalled words was found.
The aim of the study was to clarify whether mnemonic discrimination is improved by an acute bout of moderate-intensity aerobic exercise [63]	21 healthy young adults (mean age 20.5 ± 1.4 years, 10 females), without histories of neurological or psychiatric disorders. All participants had normal or corrected-to-normal vision, and normal colour vision.	In this study moderate intensity is defined as 40–59% of V̇O_2_ peak, as established by the American College of Sports Medicine (ACSM) [67]. The activity was performed by a recumbent ergometer. Mnemonic task: the participants were first shown 196 pictures of everyday objects and asked, for each of them, whether it was an indoor or an outdoor item. Then they were asked to identify by pressing a button, in the second group of 256 items, which were ‘previously seen’, ‘similar but not identical’ or ‘not previously seen’.	**The lure discrimination index (LDI) for high-similarity items was higher after 10 min of moderate aerobic exercise** than in resting controls, thus suggesting that a bout of acute aerobic exercise could improve pattern separation, that seems to rely on the dentate gyrus (DG) in humans.
The aim of the analysis was to search the literature, looking for evidence of chronic PA effects on mental health in children and adolescents [58].	Review articles reporting chronic physical activity and at least one mental health outcome (i.e., depression, anxiety/stress, self-esteem and cognitive functioning) in children/adolescents. Reviews chosen: 4 papers on the evidence concerning PA and depression; 4 for anxiety; 3 for self-esteem; 7 for cognitive functions.	Analysis based on data collected from PubMed, SPORTDiscus, PsychINFO, Web of Science, Medline, Cochrane Library, and ISI Science Citation Index, by using search terms related to the variables of interest (e.g., sport, exercise, physical activity) and mental health outcome variables (e.g., depression, anxiety, self-esteem, cognitive functioning).	**Associations between PA and mental health in young people (Tables 1–4 in Ref. [58]) is evident, but the effects are small-to-moderate, probably because of weakness of the research designs.** Small but consistent association between sedentary time and poorer mental health is also evident.
The aim of this systematic review was to find out studies elucidating the relationship between aerobic PA and children’s cognition, academic achievement, and psychosocial function [60]	Studies analysed concerned interventions of aerobic PA in children younger than 19 years. Only randomized control trials that measured psychological, behavioural, cognitive, or academic outcomes were included.	The review was performed using MEDLINE, Cochrane, PsycINFO, SPORTDiscus, and EMBASE. Additional studies were identified through back-searching bibliographies.	**Aerobic PA is positively associated with cognition, academic achievement, behaviour, and psychosocial functioning outcomes.** **More rigorous trials, however, required for deducing detailed relationships.**
Systematic review and meta-analysis of studies concerning associations between PA/sedentary lifestyle and mental health. Meta-analyses were performed in randomized controlled trials (RCTs) and non-RCTs (i.e., quasi-experimental studies) [64]	Studies published from January 2013 to April 2018. Studies were included if they comprehended PA or sedentary behaviour data and at least one psychological ill-being (i.e., depression, anxiety, stress, etc.) or psychological well-being (i.e., self-esteem, optimism, happiness, etc.) outcome in pre-schoolers (2–5 years of age), children (6–11 years of age) or adolescents (12–18 years of age).	Analysis based on data collected through a systematic search of the PubMed and Web of Science databases by two independent researchers. A narrative synthesis of observational studies was conducted.	**PA improves adolescents’ mental health, but additional studies are needed to confirm the effects of PA on children.** Findings from observational studies, however, suggest that promoting PA and decreasing sedentary behaviour might have a protecting effect on mental health in both children and adolescents.

**Table 2:** Effects of PA on circulating BDNF levels. In the first six rows, single studies have been reported, while the last two rows refer to reviews/meta-analyses. In the “Conclusion” column the main results of the analyses, as well as a few comments on them, are given.

Protocol/Aims [Ref]	Subjects/Studies Included	Methods of Analysis	Conclusions
The aim of the study was to test the effects of two high-intensity exercise protocols, already known to improve cardiovascular health, to also affect BDNF levels [103]	Experiment 1: 8 men (average age: 28 years) Experiment 2: 21 men (average age: 27 years) Both experiments included: -high-intensity interval-training (HIT), at 90% of maximal work rate for 1 min, alternating with 1 min of rest; -continuous exercise (CON), at 70% of maximal work rate. Both protocols lasted 20 min.	Experiment 1: serum [BDNF] was measured at 30 min before starting the exercise, at 0, 6, 10, 14, and 18 min during the exercise, and at the end of the exercise (20 min). Experiment2: Serum BDNF was measured only at the beginning (0 min) and at the end (20 min) of the experiment. BDNF was evaluated by an enzyme-linked immunoassay (ELISA).	-**Similar BDNF kinetics were observed in both protocols, with maximal BDNF level reached toward the end of training;** **-Both protocols (CON and HIT) significantly increased BDNF, with HIT more effective** **Shorter bouts of high-intensity exercise are slightly more effective** than continuous high-intensity exercise for elevating serum BDNF. Moreover, 73% of the participants preferred the HIT protocol Thus, the authors suggest that the HIT is an effective and preferred intervention for elevating BDNF and potentially promoting brain health.
The aim of this analysis was to study the possible relationship between exercise intensity, memory, and BDNF [104]	16 young subjects (average age: 23 years): 9 men and 7 women	3 exercise sessions at different intensities relative to ventilator threshold (Vt) (VO2max, Vt − 20%, Vt + 20%). Each session lasted approximately 30 min. Following exercise, the Rey Auditory Verbal Learning Test (RAVLT) was performed to assess short-term memory, learning, and long-term memory recall. 24 h later, the participants completed the RAVLT recognition trial, to evaluate another measure of long-term memory. Blood was drawn before exercise, immediately post-exercise, and after the 30-min recall test. Serum BDNF was evaluated by ELISA.	Long-term memory as assessed after the 24-h delay differed as a function of exercise intensity: the largest benefits were observed with the maximal intensity exercise. **BDNF significantly increased in response to exercise.** However, **no difference was noticed in relation to exercise intensity**. Similarly, no significant association was found with memory. The authors suggest that “future research is warranted so that we can better understand how to use exercise to benefit cognitive performance”.
The aim of the study was to compare basal- and post-exercise- levels of circulating BDNF, in comparison with cognitive training and mindfulness practice [105]	19 healthy subjects (age: 65–85 years)	Exercises: (1) physical aerobic exercise at a moderate level, using a Swedish version of the EA Sports Active 2™ program on a Microsoft Xbox360™ game console connected to a Microsoft Kinect™ accessory and an ordinary TV set; (2) cognitive training through a computerized working memory training program; (3) mindfulness practice through the use of the Mindfulness App (http://www.mindapps.se/themindfulnessapp/). Each program lasted 35 min. All the participants went through all the three training programs, in a random sequence. Serum BDNF was evaluated by ELISA.	**Exercise caused a significant increase in BDNF levels.** Moreover, in the same subject, **a single bout of exercise had a significantly higher impact on serum BDNF levels** than cognitive training and mindfulness practice. However, considerable variability of BDNF responses was found when comparing different subjects.
The aim of the study was to compare the effect of ‘open-skill’ with ‘closed-skill’ exercise (as defined in terms of predictability of context situations) on BDNF production [102]	20 adult males: all subjects participated in both closed (running) and open (badminton) skill exercise sessions, in counterbalanced order on separate days. Exclusion criteria: - cardiovascular disease, diabetes, history of neurological problems, pre-existing injuries, smoking or intake of recreational drugs; hearing or vision problems.	Exercise sessions: −5 min of warm-up exercises, −30 min of running or badminton. Exercise intensity: 60% of the heart rate reserve level (HRR) During each session, venous blood **samples were obtained immediately before and after exercise**. Serum BDNF was evaluated by ELISA. Cognitive performance was also evaluated by a modified form of the task-switching paradigm, and controlled via the Neuroscan Stim software.	**Badminton exercise resulted in significantly higher serum BDNF levels relative to running.** This study provides interesting evidence in support of the benefits of open-skills exercise on BDNF production and executive function.
The aim of the study was to analyse the effect of aquarobic exercise on serum irisin and BDNF levels [106]	26 elderly women: Control group: 12 subjects Exercise group: 14	Exercise sessions: 16-week aquarobic exercise program, including two sessions a week. Each session lasted for 60 min: −10 min of warm-up, −40 min of exercise, −10 min of cool. Serum irisin and BDNF levels were evaluated (three times in the exercise group and two times in the control group) by ELISA.	**Aquarobic exercises improve the serum irisin and BDNF levels.**
The aim of this study was to evaluate the effect of long-term exercise on memory and biomarkers related to cognition and oxidative stress, in healthy middle-aged subjects [107]	68 healthy men: Group 1: 21 young sedentary subjects (age: 17–25 years); Group 2: 16 young trained subjects (age: 18–25 years), Group 3: 25 middle-aged sedentary subjects (age: 47–67 years) Group 4: 24 middle-aged trained subjects (age: 46–68 years). Exclusion criteria: -history of severe disease, pain, cognitive deficiencies, head trauma. -use of neuroactive or psychoactive drugs or antioxidants.	Comparison of the BDNF levels in the four groups was performed by a two-way ANOVA. The effect of PA on cognitive abilities was evaluated by a combination of neuropsychological tests, among which: the Trail Making Test, Part A and Part B, the Wechsler Adult Intelligence Scale IV Digit Span Subtest32, the Stroop Interference Test31, the Computerized tests from Cambridge Neuropsychological Test Automated Battery (CANTAB software, Cambridge Cognition, UK), and the Free and Cued Selective Reminding Test (FCSRT)33 Serum BDNF levels were measured by ELISA.	The Free and Cued Immediate Recall tests showed significant improvements in memory in the middle-aged trained individuals when compared to the sedentary ones. **A significantly lower resting level of serum BDNF (and plasma Cathepsin B) was observed in both trained groups**. **In particular, BDNF and CTSB levels were inversely correlated with weekly hours of exercise.**
The aim of the analysis was to find out any exercise-dependent correlation between BDNF concentration and aerobic metabolism in healthy subjects [100]	Studies were included when they reported BDNF analysis before and after at least one session of exercise. Total studied included: 20	Analysis based on papers collected from PubMed, Scopus, and Medline databases.	**PA-induced BDNF increase is related to the amount of aerobic energy required in the exercise, in a dose-dependent manner.**
Protocols: -Preferred Reporting Items for Systematic Reviews and Meta-Analyses Protocols (PRISMA-P) -Cochrane Handbook of Systematic Reviews of Interventions [108]	Inclusion criteria: studied conducted on adolescents trained with different exercise protocols, and including evaluations of pre- and post-intervention BDNF levels.	Data derived from PubMed, EMBASE, Scopus, ScienceDirect, Web of Science, SPORTDiscus, the Cochrane Central Register of Controlled Trials (CENTRAL), and CINAHL.	**The results show that BDNF levels increase after interventions, regardless of whether the aerobic exercises were acute or chronic.**
